# Development of a Resource Guide to Support the Engagement of Mental Health Providers and Patients With Digital Health Tools: Multimethod Study

**DOI:** 10.2196/25773

**Published:** 2021-04-22

**Authors:** Gillian Strudwick, David McLay, Brian Lo, Hwayeon Danielle Shin, Leanne Currie, Nicole Thomson, Éric Maillet, Vanessa Strong, Alanna Miller, Nelson Shen, Janis Campbell

**Affiliations:** 1 Centre for Addiction and Mental Health Toronto, ON Canada; 2 University of Toronto Toronto, ON Canada; 3 Dalhousie University Halifax, NS Canada; 4 University of British Columbia Vancouver, ON Canada; 5 University of Sherbrooke Longueuil, QC Canada; 6 Memorial University St. John's, NL Canada; 7 McGill University Montreal, QC Canada

**Keywords:** digital health, mental health, psychiatry, COVID-19, nursing informatics, health informatics

## Abstract

**Background:**

As mental illness continues to affect 1 in 5 individuals, and the need for support has increased during the COVID-19 pandemic, the promise of digital mental health tools remains largely unrealized due to a lack of uptake by patients and providers. Currently, most efforts on supporting the uptake of digital mental health tools remain fragmented across organizations and geography. There is a critical need to synthesize these efforts in order to provide a coordinated strategy of supporting the adoption of digital mental health tools.

**Objective:**

The specific aim of this project is to develop a web-based resource document to support the engagement of mental health providers and patients in the use of digital mental health tools.

**Methods:**

The web-based resource was developed using a multimethod approach. A grey literature review was conducted in 2019 to identify relevant toolkits that are available in the public domain. This was supplemented with an environmental scan where individuals with expertise in the development, acquisition, implementation, and evaluation of digital mental health tools were invited to contribute additional tools or documents not identified in the grey literature search. An engagement workshop was held with stakeholders to explore how the resource document should be developed and delivered. These findings were collectively used to develop the final iteration of the resource document.

**Results:**

Based on a gray literature review and environmental scan with 27 experts, 25 resources were identified and included in the resource guide. These resources were developed for patients and providers by organizations from 5 countries. An engagement workshop was held with 14 stakeholders, and barriers related to cultural sensitivity, sustainability, and accessibility of the toolkit were identified. The final iteration of the resource document was developed by the research team using findings from the gray literature review, environmental scan, and engagement workshop. The contents of the 45-page resource guide are directed at mental health care providers, administrators, and patients (inclusive of families and caregivers).

**Conclusions:**

The use of a multimethod approach led to the development of a resource guide that builds on existing evidence on digital mental health tools and was co-designed with stakeholders and end-users. The resource guide is now publicly available online for free and is being promoted through digital health and mental health websites. Future work should explore how this document can be integrated into clinical care delivery and pathways.

## Introduction

Mental illness continues to be a global challenge, particularly during the COVID-19 pandemic [1]. Even prior to the pandemic, mental illness affected 1 in 5 individuals in the United States [2]. Unfortunately, with fewer than 50% of individuals receiving treatment for their mental health issues, mental illness remains a top contributor of disability in many countries including the United States [3] and Canada [4]. This increasing and unprecedented demand has spurred great interest in the use of telehealth or other digital tools during the current pandemic, highlighting new opportunities for improving access to health care through digital technologies [5].

Digital mental health interventions, such as mobile apps, have been advocated by organizations, including the American Psychiatric Association (APA) [6] and the Health and Aging Department of Australia [7], as promising tools to support the current challenges in mental health service delivery. However, uptake of these tools by patients and providers remains poor [8]. In response, tools and resources for supporting the uptake of these tools into practice have recently been developed [9], yet these resources remain largely underused. Currently, most of the efforts and resources that are developed to support the adoption of digital mental health tools have been done in a piecewise effort across government organizations [10], hospitals, and mental health associations [11]. To our knowledge, there is no single source or repository where users can seek guidance on identifying relevant eHealth technologies for their needs. Given this, there is a critical need to collaborate with stakeholders and end-users in synthesizing a strong body of guidance and evidence to support digital health activities (eg, usability, user needs) and accelerate the adoption of digital tools for mental health contexts, especially during a global pandemic.

The objective of this project is to develop a comprehensive web-based resource guide to support mental health providers and patients in the selection and adoption of digital health tools through consideration of relevant factors (eg, demographics, clinical needs). The intended audiences of the guide are mental health care providers (eg, psychologists) and administrators (eg, implementation specialists) who are interested in integrating digital health tools into clinical practice, as well as people with lived experience and families or caregivers looking to use the resource to select helpful tools for their own needs. In this article, we share our approach and methodology for developing the resource document and outline how the main findings from each phase of the method informed the final development of the resource document. In addition, the implications of the resource document and challenges identified throughout the development process are discussed.

## Methods

**Figure 1:**
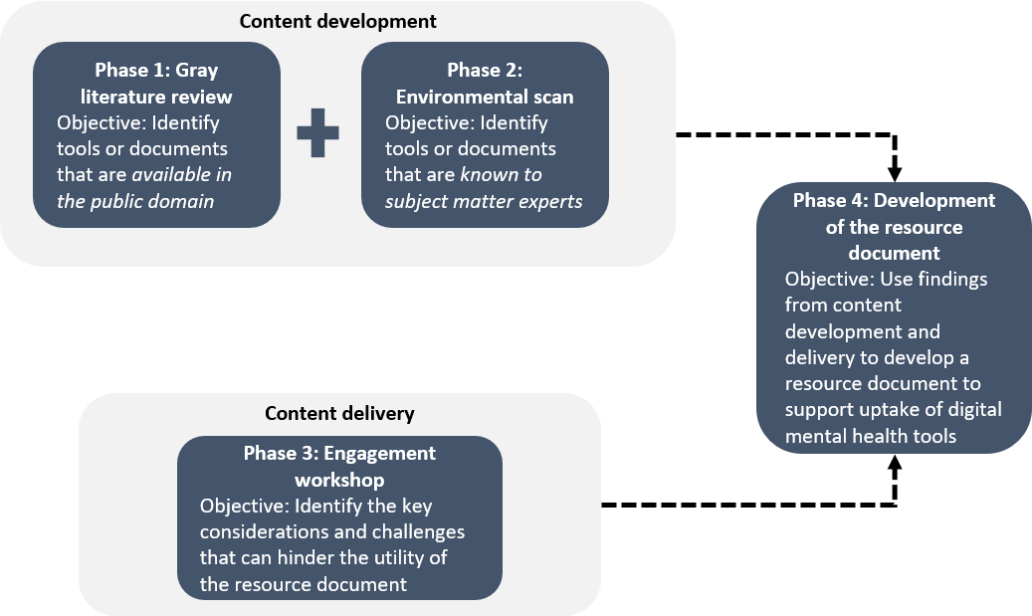
Overview of the multimethod approach for the development of the resource document.

Following guidelines of the Agency for Healthcare Research and Quality [12], we used a multimethod approach (Figure 1) to develop the resource guide. The methods include a gray literature review, an environmental scan and engagement of experts in the field, and an engagement workshop with relevant stakeholders from a variety of backgrounds and interests. The findings from these 3 sources were then used to inform the development of the final version of the resource document (Multimedia Appendix 1). A multimethod approach [13] was selected to maximize the consolidation efforts of this work and deliver the findings in a meaningful and useful resources for a diverse audience. This work spanned across all provinces in Canada.

### Phase 1: Gray Literature Review

The objective of the gray literature review [14] was to identify relevant toolkits that are publicly available in the public domain. Following best practices on gray literature review [15], one of the authors (DM) conducted Google searches using key terms related to digital mental health tools, resources, and toolkits (Textbox 1). The first 10-20 pages of the Google Search results were reviewed, and tools or documents that met the inclusion criteria were identified and included. The inclusion criteria were as follows: available in English; technologies used or referred available in Canada; practical guidance for the use of digital mental health tools in clinical care provided; a target audience of providers, clients and caregivers, or both; and having relevance or being easily adaptable to the Canadian context (eg, health care system structure, processes).

Preference was given to Canadian sources and bilingual (French and English) resources. Relevant websites from mental health organizations (eg, Canadian Mental Health Association), medical organizations or hospitals (eg, British Medical Association), patient organizations (eg, The Mental Elf), and governmental organizations (eg, US Department of Health and Human Services) were also included. Tools of documents were excluded for one or more of the following reasons: they were more than 3 years old; they were digital mental health tools (eg, mental health apps, telemedicine portals); information was intended for policymakers, industry, or other audiences outside of providers, clients, or caregivers; they were academic or research articles; the tools had significant contextual information (eg, legal context or policy context) that rendered the information irrelevant for the Canadian context.

Blogposts or other lists (usually of apps) were also excluded due to a concern for the information being outdated. Included tools or documents were then catalogued using a Microsoft Excel spreadsheet for analysis. Relevant information related to the scope and utility was extracted from each tool or document. A content analysis [16] was used to understand the characteristics of the included tools and documents.

**Search strategy for gray literature review.**

**Gray literature search strings**

• (electronic OR digital OR mobile) AND “mental health” AND (tool OR resource or e-tool OR e-resource OR toolkit OR app OR web)

• (electronic OR digital OR mobile) AND patient AND (tool OR resource OR e-tool OR e-resource OR toolkit OR app OR web)

• digital mental health tool

• digital tools to help my mental health

**Phase 2: Environmental Scan**

The purpose of the environmental scan was to identify relevant documents and tools that currently exist and are used in the field. To maximize the impact of the environmental scan and the number of documents and tools found, experts in Canada and the United States were identified using a snowball sampling approach through the professional networks of the project team and those who published, conducted research, or worked in the field. Individuals who were knowledgeable across various digital health activities (eg, implementation, evaluation, design) were eligible to participate. Experts were contacted via email and telephone by the project team. Each expert was asked if they were aware of any tools or documents relevant to guide the uptake of mental health tools in the delivery of mental health care. These tools or documents were added to the list from the gray literature review (phase 1) and screened using the same inclusion and exclusion criteria. Content analysis [16] was also used to characterize the identified documents.

### Phase 3: Engagement Workshop

We conducted an engagement workshop to increase the relevance and use of our research findings in practice [17]. Based on the environmental scan and literature review, a 1-day engagement workshop was held in January 2020 to gather information on the design and use of the resource guide. The engagement workshop was based on the Theory of Inventive Problem Solving (TRIZ) framework, which was developed in the 1960s to solve problems through the development of innovative and creative solutions [18]. Stemming from the principle of identifying the conceptual issue, it has become the basis for developing a conceptual solution that guides the arrival of the actual solution [18]. In contrast to traditional approaches, the TRIZ is said to guide the development of solutions by focusing on the contradictions that arise between the ideal and real system, and identifying solutions that close this gap [18]. Stakeholders, with diversity across roles, perspectives, geographies, and gender, were invited to participate in the engagement symposium. Individuals were eligible to participate if they had interest or experience with digital mental health tools. The inclusion criteria were kept broad to ensure we maximized the diversity among participating stakeholders. The structure of the workshop was completed using a World Café style [19], which is a structured approach to gather feedback from a large audience. The engagement workshop begins with asking participants to consider elements that would make the worst resource guide ever, and what approaches can be leveraged to prevent this from happening. Following this discussion, participants were invited to comment on what would be the critical elements that are relevant for an excellent resource. After each participant discussed these questions within the smaller group, a larger discussion was conducted before the engagement workshop was concluded. In the afternoon, participants were asked about current approaches for seeking information on digital mental health tools and how this resource document may be implemented to address current unmet needs. Challenges related to the uptake of the resource document were also discussed. Notes were taken by a member of the research team (JC) and were consolidated using a content analysis [16] approach.

### Phase 4: Development of the Resource Guide

In order to develop the resource guide, the aforementioned efforts were consolidated by the research team. Foremost, after screening of the identified toolkits from the gray literature scan and environmental scan, a member of the research team (DM) consolidated and organized the list of resources based on the purpose and description provided by each toolkit. Each toolkit was also characterized by the intended audience, format, scope, language, and country of origin. The findings from the engagement workshop were then used by the research team to refine the draft of the resource document to a format and delivery that aligned with the need of stakeholders.

## Results

The gray literature review and environmental scan led to the identification of 25 resources that were deemed relevant for the resource document. The engagement workshop, which was conducted with 14 participants, was used to inform the development of the resource document (Multimedia Appendix 1) [20]. A detailed description of the findings from each phase of the development (Figure 1) can be found in the next section.

### Phase 1: Gray Literature Review

The gray literature review was conducted in September 2019, and a total of 19 resources were identified. Most of the identified resources from this phase of the project were websites or blog posts that contained a collection of apps (n=9). Other types of resources included app rating frameworks (n=3), implementation guides for clinicians (n=2), evaluation tools (n=1), electronic health record–related comic strips (n=2), and social media and info guides (n=2).

Of the 19 resources that were identified from the gray literature review, only 11 (60%) of the tools or documents met the inclusion criteria and were summarized for the final iteration of the toolkit. These included the HITEQ (Health Information Technology Evaluation, and Quality Center) Health App Decision Tree [21], the “e-Mental Health in Practice” document [22] from the Black Dog Institute, and patient information guides [23] from the National Health Service in the United Kingdom.

### Phase 2: Environmental Scan

A total of 27 experts from Canada and the United States participated in the environmental scan. The demographics of the experts are outlined in Table 1. These experts have administrative, clinical, or research roles in mental health and either actively participate or have experience in activities (eg, development, implementation) related to digital mental health tools. There was representation across many provinces in Canada, with most participants being from Ontario (n=10). In terms of organization, most participants had affiliations with academic and government organizations. From the 42 tools or documents that were suggested by participants of the environmental scan, a total of 14 tools or documents met the inclusion criteria and were included in the final resource document.

**Table 1:** Demographic characteristics of participants in the environmental scan.

Characteristic		Number of participants (N=27), n (%)
**Province/location**		
	British Columbia	1 (4%)
	New Brunswick	2 (8%)
	Nova Scotia	8 (30%)
	Ontario	10 (37%)
	Prince Edward Island	2 (8%)
	Quebec	1 (4%)
	Saskatchewan	1 (4%)
	Outside of Canada	2 (8%)
**Organizational affiliation**		
	Academic institution	10 (37%)
	Government	9 (33%)
	Hospital	3 (11%)
	Nonprofit organization	5 (19%)

### Characteristics of Resources

The gray literature review (phase 1) and environmental scan (phase 2) led to the identification of 25 resources in our web-based resource guide. Among the 25 resources, 9 resources (36%) provided ratings or reviews of digital mental health tools, and 3 resources (12%) provided guidance on the implementation of these technologies. Additionally, there were 4 resources (16%) that were tools for patients, 6 resources (24%) for clinicians, and 3 (12%) resources designed for both patients and clinicians.

In terms of the resources identified, most were developed in Canada (n= 10) and the United States (n=10), with other resources being from the United Kingdom (n=2), Australia (n=2), and New Zealand (n=1). Only resources developed in Canada were found to be available in French. In addition, only 60% of the resources (15/25) were developed or updated in 2018 and later. The latter resources do not have an updated date or were last updated before 2018. Most resources were developed collaboratively with private or not-for-profit organizations (n=15), academic groups (n=6), provider associations like Canadian Medical Association (n=5), health care organizations (n=2), and governments (n = 2). Likewise, funding for the development of the resource originated from not-for-profit organizations (eg, One Mind), health service organizations (eg, Ministry of Health of New Zealand), government-funded organizations (eg, Ontario Telemedicine Network), provider organizations (eg, British Medical Association), and academic institutions (eg, University of Chicago).

In terms of the audience, identified resources included content relevant to patients (n=10) and clinicians (n=20). In particular, 15 resources had clinician-specific resources, 5 resources contained patient-specific resources, and 5 resources had content for both populations. Some resources indicated a specific audience, such as primary care or general providers (n=2), frontline workers (n=2), physicians (n=1), medical school students (n=2), and researchers or app developers (n=1). Some resources targeted a specific mental health condition (eg, depression), while 13 resources focused broadly on mental health and e-mental health technologies.

As per the typology outlined by the Mental Health Commission of Canada (MHCC) [24], 5 resources reviewed and rated “computerized treatments, resources & apps,” and 4 resources provided frameworks to conduct evaluations and reviews of these technologies. In addition, 3 resources provided implementation frameworks and guidance on integrating these technologies into clinical environments. These implementation frameworks were not limited to a single category within the typology.

The report from the MHCC [24] suggested that apps should disclose information related to the evidence base, cultural appropriateness, and gender responsiveness. For the 5 resources that provided app reviews, 4 of the sites included supporting evidence and 3 of the sites outlined privacy information about the apps. Although all tools are free to access, some tools reference apps that have a cost requirement, and these requirements are indicated in some tools (eg, Psyberguide). At last, only 2 of the sites provided data (eg, Practical Apps) about usability and user experience.

### Phase 3: Engagement Workshop

A total of 14 participants from mental health organizations across Canada took part in the engagement workshop that was facilitated by 6 members (GS, DM, LC, HDS, AM, and JC) of the research team. The demographics of participants and facilitators in the engagement workshop are outlined in Table 2.

**Table 2:** Demographic characteristics of participants in the engagement workshop.

Category			Number of participants including facilitators (N=20), n (%)
**Gender**			
	Female	16 (80%)	
	Male	4 (20%)	
**Province**			
	British Columbia	1 (5%)	
	New Brunswick	2 (10%)	
	Newfoundland	1 (5%)	
	Nova Scotia	2 (10%)	
	Ontario	13 (65%)	
	Quebec	1 (5%)	
**Role/contribution (multiselection)**			
	Clinician (eg, nurse, psychologist)	13 (65%)	
	Graduate trainee	4 (20%)	
	Indigenous perspective	1 (5%)	
	Person with lived experience	2 (10%)	
	Research personnel/expert	4 (20%)	

In the first part of the exercise, the research team asked the participants, “What would make the worst toolkit ever?” Respondents suggested that accessibility barriers were an important consideration. Examples of accessibility barriers included a lack of “searchability” and poor user-friendliness of the resource guide itself. A document with too much text and use of jargon would make it difficult for the end-user to effectively integrate it into practice. In addition, respondents highlighted the need for making the resource a “living document” that does not contain outdated information and broken links. It was further noted that resources that are not culturally sensitive and not trauma-informed may also be dangerous for the end-user and can impede the value of the resource. Other factors discussed included a lack of a dissemination plan, discussion on privacy issues, and the absence of patients, families, or the community in the development of the resource guide.

The subsequent discussion aimed to address the challenges identified by exploring the questions “How could we prevent this from happening?” and “What would the best possible toolkit look like?” Participants made several suggestions including a focus on evidence-based development of the document, co-design with the audience, and development of a postdevelopment sustainability plan. It was indicated that the evidence-based development should be inclusive of the views and perspectives of intended end-users and include open-source links for readers to explore if they are interested. It was further suggested that the methodology of the resource guide be transparent in the resource document. Participants explained that the content of the resource document should also be inclusive of the different learning styles of individuals and manage the expectations of the reader (ie, relatively new field). With regard to the postdevelopment sustainability plan, participants suggested a “review cycle” where the materials would be revisited after a certain period of time to ensure up-to-date content. In addition, a follow-up/feedback loop with participants was encouraged to allow for continuous improvement of the resource document.

The afternoon session of the workshop focused on dissemination of the resource document. When participants were asked where they seek information on digital mental health tools, a variety of academic (eg, school) and professional (eg, regulatory college) organizations were listed. Other approaches included conferences, word of mouth, and the intranet of their employer. With regard to the challenges of implementing the resource document for uptake of digital mental health tools, there were concerns on the definition of the “toolkit” and who the target audience is. There was also discussion on how the scope of the toolkit may not be compatible with current structure of care systems. For example, some clinicians may not have a choice in deciding which tools would be made available to the patient, and the process would require engaging stakeholders across project management, clinical services, and privacy domains. It was also unclear if this resource document would be based on current principles of mental health care, such as the stepped care model [25]. Finally, some participants gave suggestions on delivering the contents of this document in other formats, such as a webpage as opposed to a static PDF document [20].

### Phase 4: Development of the Resource Document

The findings from phases 1-3 of this project were used to develop the final iteration of the resource document (Multimedia Appendix 1) by the research team. The 45-page resource document [20] begins with background information on digital mental health tools and how various tools could be used to support the mental health needs of an individual. A set of questions were also developed to help guide a client’s decision on whether digital mental health tools are appropriate for their needs. The list of resources identified from phase 1 and 2 was then summarized in a chart by audience, format, language, country of origin, and whether the toolkit is specific to mental health. A summary of each of tool or document is subsequently provided. This summary includes additional information such as whether internet connection is required, if data is collected on the user, and the suitability of the resource for use during interaction with a client. The resource document concludes with a high-level overview of the project methodology.

Moreover, many suggestions and concerns from the engagement workshop (phase 3) were incorporated in the development of the final version of the resource guide. For example, the language used throughout the document was reflective of the suggestions of the stakeholders (eg, neutral, welcoming, and free of jargon) and brief instructions were provided at the beginning of the resource document to orient the end-user on usage of the document. The guide was also optimized for the search functionalities of the application.

## Discussion

### Principal Findings

Although digital mental health tools have gained significant traction and interest from patients, caregivers, family members, providers, and mental health organizations [26], uptake and integration of these technologies remain fairly poor across mental health care [8]. From the gray literature review (phase 1) and environmental scan (phase 2), a total of 25 resource guides that were relevant in supporting the uptake of digital mental health tools were identified. These resource guides were developed by various health care and mental health care organizations and targeted both patients and clinicians. Feedback on the delivery of these findings were identified from the engagement workshop with 14 participants. These findings collectively informed the development of the final resource document [20], which can be found in Multimedia Appendix 1.

In our experience, the use of a multimethod approach [13] provided a solid foundation for developing a document that aligns with the needs of the clients, caregivers, and end-users. Of the 25 resources that were identified, there was a relatively even spread of resources from the gray literature review (phase 1) and the environmental scan (phase 2). This demonstrates the importance and value of both sources as part of a comprehensive synthesis of tools or documents relevant to digital mental health tools. However, the absence of guidance on the delivery of the content can jeopardize the success of the project and lead to products that are not compatible with the needs of end-users [27]. Our engagement workshop (phase 3) was instrumental in engaging patients, clinicians and other relevant stakeholders in addressing this gap in guidance [27]. The application of the World Café [19] approach facilitated the consensus of opinions and perceptions throughout the workshop and provided great insight into how the resource document should be best delivered. Thus, the multimethod approach [13] is a valuable approach in consolidating the knowledge from each source to develop a relevant and timely resource document that is applicable for a variety of audiences.

This paper introduces a web-based resource guide [20] that our research team designed to foster the engagement of mental health patients and providers with digital mental health tools. This resource guide [20] is now publicly available for free and is expected to be used by both patients and practitioners in supporting the uptake of digital mental health tools. Patients and their caregivers may use this document to choose appropriate resources to guide the selection of a suitable app to meet their needs and requirements. In addition, using the questions that are listed on pages 9-11 of the resource document [20], individuals can also examine if digital mental health tools are appropriate and suitable for their needs, or if other (eg, in-person) interventions are necessary. Similarly, this resource guide will help providers and clinicians become acquainted and knowledgeable about the use of digital mental health technologies. In particular, providers may consider this resource document in speaking to patients and family members about the use of digital mental health tools as part of care [28]. Providers may also consider using this document as a means to guide the conversation and planning of which tools should be used and in what manner [29]. At a broader level, health care administrators may also use this newly developed resource to develop proper training and support for providers interested in using digital health tools in their practice.

During the development of the document, a number of evidence gaps were identified. Chiefly, the identified resources included in the resource guide fail to cover many of the technologies outlined in the MHCC typology [24]. As most of the published resources focused on mobile health apps, there is a lack of resources for other technologies such as virtual reality technology and robots [30-32]. Future work that focuses on the uptake of these emerging technologies would be useful [8]. Additionally, most of the resources are available in English only. Making the identified resources available in other languages, such as French, would be helpful [33], particularly given the abundance of French-speaking people in Canada. Similarly, none of the identified tools encompass other cultures, including indigenous perspectives, or address cultural appropriateness. Furthermore, given the emerging crisis of caregivers, future tools should explore the role of e-mental health technologies to support the needs of caregivers [34].

Although this resource document was developed prior to the COVID-19 pandemic, we expect that it will continue to be of value for supporting the ongoing mental health needs and demands during and beyond the pandemic. However, it is important to note that the pandemic has greatly accelerated the uptake of some digital mental health tools [35]. For example, many organizations have converted their delivery of outpatient or ambulatory care to telemental health visits (eg, using Zoom or Microsoft Teams) [28]. Some of these changes in practice have also led to the use of digital mental health tools, such as patient portals and mobile apps [36]. As part of the postdevelopment sustainability plan, it would be of value to synthesize the recent outputs from the use of digital mental health tools during the COVID-19 pandemic [37] to the current resource document.

### Limitations

Although this resource guide has been developed with extensive input from gray literature and experts in the field, it has yet to be integrated into clinical workflows and refined by providers with experience using this resource with patients [8]. Additionally, the identification of relevant resources was limited to those available in the English language. Experts who participated in the engagement workshop and environmental scan were also all from North America. Exploring resources of other languages and consulting experts from other countries (eg, the United Kingdom) may provide insight into novel resources not identified here. Although, the findings from the engagement workshop were derived from a technique used to support consensus (World Café) [19], further validation of the findings (eg, member checking) [38] was not conducted with the participants of the group or with other participants. Moreover, the participants were not engaged during the review of the final iteration of the resource document. Thus, there is a need to evaluate the efficacy of the toolkit. Currently, it is unclear how the document may impact the uptake of digital mental health tools [8,26,39] (eg, patient–clinician relationship [40-44]), and examining the analytics and usage of these tools [45] may provide insights into this evidence gap.

### Future Directions

This document is the product of a careful and meaningful synthesis of resources that encourage uptake of digital mental health tools, and future work should explore how these tools can be or have been integrated into clinical care pathways for mental health conditions (eg, depression). This may involve promoting and sharing the resource document across organizations that may be interested in the uptake of digital mental health tools. This may include the identified recommendations from the engagement workshop on expanding the delivery of the resource document to web-based approaches (eg, website, mobile app). It would also be useful to validate the findings from the resource document (eg, with other similar toolkits or documents) and to examine the efficacy of this resource document in addressing barriers and opportunities of digital mental health tools [38]. This can be conducted using a mixed methods approach [46] to incorporate both measurable outcomes and user experience. Moreover, at the solution level, identifying strategies to enhance uptake of emerging digital mental health tools is warranted. There remains a need to examine factors (eg, gamification [47]) that may relate to the engagement with digital mental health tools [8]. Finally, with regard to the recent events of the COVID-19 pandemic [35,37], it would also be useful to explore additional work and guidelines that are being developed during the pandemic and their impact on engagement with digital mental health tools, particularly concerning virtual care and telemental health [48,49].

### Conclusions

This paper describes the development of a web-based resource guide that we designed to guide the uptake of digital mental health tools into the clinical environment through a multimethod approach. The document, which is available online for public use, includes a number of resources to guide the selection, implementation, and evaluation of digital mental health tools. Although these resources cover many objectives and audiences, there are disproportionately fewer resources available for emerging technologies like virtual reality. Moreover, the lack of resources designed for caregivers warrants further research. There is also a critical need to ensure that resources are inclusive of the needs of diverse cultures, including the First Nations, Inuit, and Métis people of Canada. Finally, future work should explore how this resource guide can be adopted and integrated into clinical environments.

## Appendix

Multimedia Appendix 1 Final version of the resource document.

## Abbreviations

APA: American Psychiatric Association
HITEQ: Health Information Technology Evaluation, and Quality Center
MHCC: Mental Health Commission of Canada
TRIZ: Theory of Inventive Problem Solving
