# Supplementary material for: Development of a Resource Guide to Support the Engagement of Mental Health Providers and Patients With Digital Health Tools: Multimethod Study
Source: J Med Internet Res. 2021 Apr 22;23(4):e25773. doi: 10.2196/25773 (PMC8103299; doi:10.2196/25773)
Supplement: Multimedia Appendix 1 [file jmir_v23i4e25773_app1.pdf]

Digital Mental Health Tools: Resources to Support Mental Health Clinical Practice

April 2020

camh

Canada Health Infoway

Digital Mental Health Tools: Resources to Support Mental Health Clinical Practice
Copyright © 2020, Centre for Addiction and Mental Health

No part of this work may be reproduced or transmitted in any form or by any means electronic or mechanical, including photocopying and recording, or *by any information storage and retrieval system without written permission from the publisher—except for a brief quotation (not to exceed 200 words) in a review or professional work.*

Disclaimer: Information in this document is not to be considered a recommendation or advice. Users relying on this information do so entirely at their own risk. Neither the authors, editors or publishers, nor any partners or funders, may be held liable for damages of any kind that may result from the use or misuse of any such information. If expert assistance is needed, the services of a competent professional should be sought. The views xpressed herein do not necessarily reflect the policies or opinions of authors, partners or funders.

Suggested citation: Strudwick, G., McLay, D.W., Currie, L.M., Thomson, N., Maillet, E., Campbell, J., Miller, A., Shin, H.D., Strong, V. (2020). Digital Mental Health Tools: Resources to Support Mental Health Clinical Practice. Centre for Addiction and Mental Health: Toronto, ON. This document is available at camh.ca/digitalMHtoolsresource

Project commissioned by Canada Health Infoway
This document was produced by CAMH Public Affairs
www.camh.ca

6142 / 04-2020

CONTENTS

ii ACKNOWLEDGMENTS

ii PROJECT TEAM

1 Introduction

8 Is this the right tool for you? Questions for clients

13 Resource chart

15 Resource summaries

33 Project summary

36 REFERENCES

38 APPENDIX 1: GLOSSARY

39 APPENDIX 2: ENVIRONMENTAL SCAN PARTICIPANTS

40 APPENDIX 3: ORGANIZATIONS INCLUDED IN GREY LITERATURE SCAN

ACKNOWLEDGMENTS

This project is a collaboration between the Centre for Addiction and Mental Health (CAMH) and Canada Health Infoway, in consultation with many stakeholders from across Canada. Thank you!

PROJECT TEAM

Gillian Strudwick, RN, PhD, CAMH, Toronto

David McLay, PhD, CAMH, Toronto

Leanne M. Currie, RN, PhD, University of British Columbia, Vancouver

Nicole Thomson, OT Reg. (Ont.), PhD, CAMH, Toronto

Eric Maillet, RN, PhD, University of Sherbrooke, Longueil

Janis Campbell, MEd, CCC-S, Memorial University of Newfoundland, St. John's

Alanna Miller, BSc, McGill University, Montreal

Hwayeon Danielle Shin, BScN, RN, Dalhousie University, Halifax

Vanessa Strong, MSc, Memorial University of Newfoundland, St. John's

Introduction

Goals and objective

The **goals** of this document are to:

- support the increased use of digital health tools in mental health clinical practice in Canada
- help health care providers to empower clients (and their caregivers) to use and benefit from digital mental health tools.

The **objective** of the document is to identify existing digital health resources, to allow users to choose and integrate appropriate resources into the client-provider interaction.

A summary of the project can be found on page 33.

Audience

Our primary audience is health care providers working in mental health in Canada who are looking for support in integrating digital health tools into their clinical practice. This includes:

- **health care providers and other providers in mental health care**, including psychiatrists, psychologists, counsellors, psychotherapists, nurses, primary care physicians, social workers, occupational therapists and peer support workers
- **administrators** wishing to increase the uptake of digital mental health tools, such as program planners, digital health implementation specialists, information management / information technology specialists and process improvement specialists.

In addition, **clients and caregivers** are the audience for several of the resources in this document. We invite clients and caregivers to use the document to identify resources that might be helpful to them.

The primary audience for this document will vary between organizations, depending on contextual factors such as size, policies and existing infrastructure. We encourage you to consult within your organization before implementing a resource listed here.

What is in this document?

This document describes 18 resources that can support the integration of digital mental health tools into the interaction between client and provider. The resources have a wide range of objectives, formats and audiences. For a concise, high-level review of the key features of each resource, see the Resource Chart (page 13).

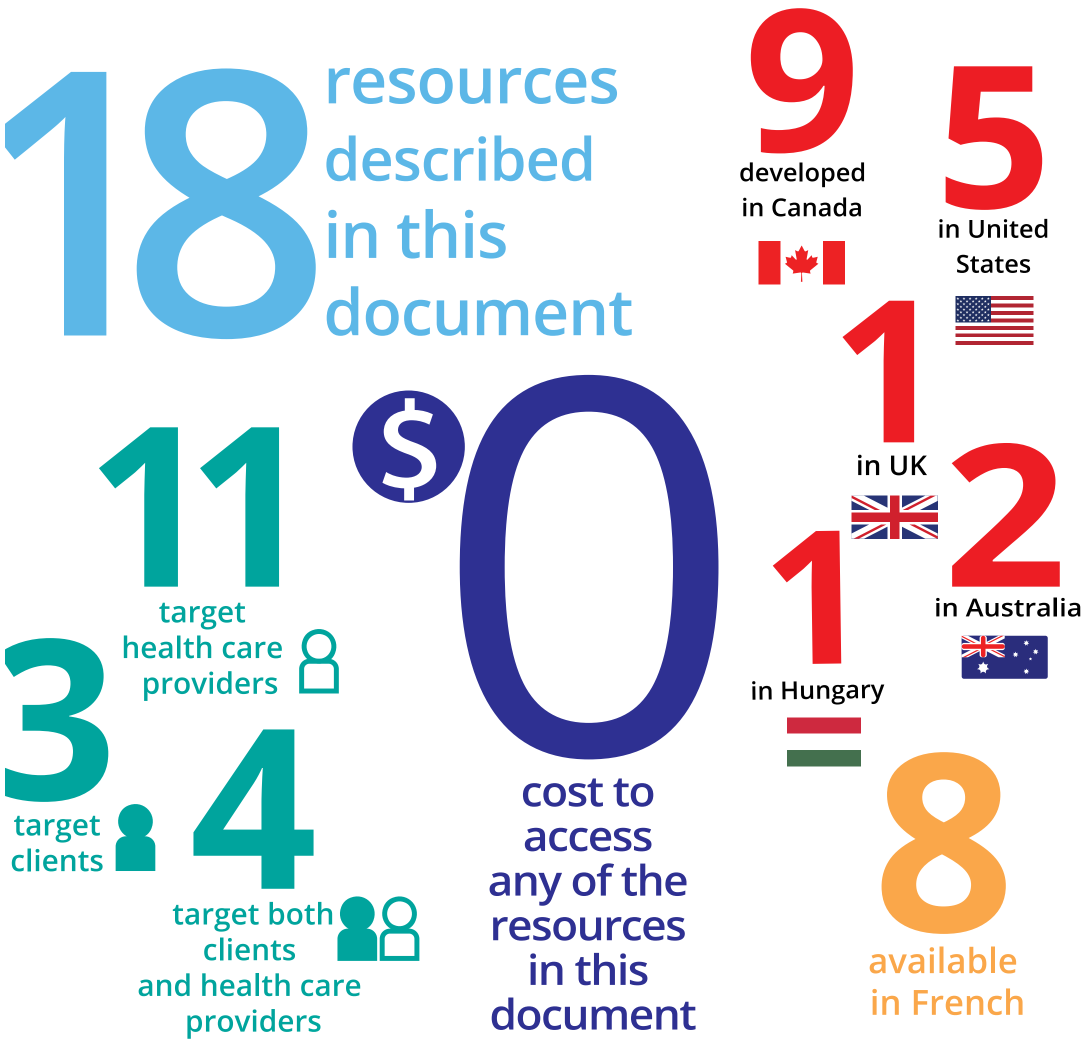

Examples of the type of resources in this document

Psyberguide

This website provides reviews and ratings of mobile apps related to mental health. The website helps providers (or clients) choose an app, but does not offer mental health support.

Mental Health, Technology and You

This client guide offers descriptions of digital mental health tools, as well as personal stories, a journey map and tips for staying safe online. The guide helps clients understand digital mental health tools and imagine how they might use them. It is not itself a tool for digital mental health care, but rather helps people understand and use digital mental health tools.

These types of resources are relatively new. Much of the focus to date has been in helping users choose and integrate apps and in implementing digital mental health programs through comprehensive guides. Many gaps remain. For example:

- No resources were found that directly address tools related to virtual reality, robots, gaming or artificial intelligence.
- No resources were found that target caregivers. More explicit attention to their unique position in supporting clients (and providers) would undoubtedly promote the uptake and use of digital mental health tools.
- In general, resources seemed not to account for cultural differences. For example, no resources were found that target Indigenous people. Given their unique and diverse cultures, this gap may do a disservice to the First Nations, Inuit and Métis people of Canada.

What this document is not

This document is **not**:

- a collection of digital mental health tools (e.g., specific apps or technologies) that can be used in the client-provider interaction; rather it describes a number of resources that can support the integration of digital mental health tools into clinical practice (see Appendix 1: Glossary [page 38] for more information).
- an expert rating or review of the resources presented; we encourage you to assess yourself whether the resource meets your needs
- updated in an ongoing way; this document was published in early 2020 and represents the state of the field at that time.

How to use this document

Finding a resource

The most direct way to access a specific resource is to:

1. Go to the Resource Chart (page 13).
2. Review the features of the resources and note the page number for the summary of the resource you're interested in.
3. Go to that page in the Resource Summaries section to learn more about the resource.
4. Follow the hyperlink or cut and paste the URL into a web browser to access the resource.

You can also browse the Resource Summaries section (page 15) in a more open-ended way.

Finally, you may search the document by using your PDF reader's search function, often accessed by **Ctrl + f**.

Using the resources

Information on how to use a given resource is often provided in the resource itself. For many resources, it is self-evident. In the summary of each resource, we provide some basic information on how to use it.

When thinking about how you might use a resource in your clinical practice, consider the discussion in the section (Is This the Right Tool for You?, page 9) of barriers and facilitating factors that influence the uptake of digital mental health tools.

Exploring the use of digital mental health tools

Advances in technology continue to revolutionize the health care system and influence the practice of health care professionals. Introducing digital tools and technology into the mental health care system has the potential to shorten wait times, increase access in rural and remote communities, and provide cost-effective access to care.[1] Research has demonstrated that many mental health care providers are aware of and ready to utilize these opportunities.

Nonetheless, several factors specific to mental health care need to be considered when implementing digital tools. One overarching consideration to keep in mind is that no single tool will work for everyone.[11] Other factors to consider are described below.

Potential uses

Enthusiasm for the use of digital tools is high among mental health care providers: in one survey, 98% of respondents said they were ready to use technology to support their clients.[2] Providers see the potential for digital health tools to increase access to mental health care and support for their clients.

There are many potential ways in which digital health tools may be used in mental health care. Here are a few examples:

- A computer-assisted client assessment or survey could be created for people waiting to see their family physician. The technology could promote self-disclosure through a non-intrusive means of prompting discussion of mental health concerns.[3]
- Incorporating technology-mediated supervision, such as a digital platform that would allow mental health providers to consult with each other on their use of digital tools, may provide the necessary support for providers to incorporate digital tools and technology in their clinical practice.[4]
- Tools to enhance face-to-face therapeutic interventions could allow therapy to extend beyond the session itself, and so contribute to treatment progress.[5]
- Digital health tools could help to personalize mental health treatment by sharing individualized plans or tracking progress.

Ethical, legal, privacy and confidentiality considerations

It is important to help clients to understand the ethical, legal, privacy and confidentiality considerations of digital tools. Areas to consider include:

- understanding the detailed risks and benefits of each tool used[5]
- the security risks and security settings of different technologies and digital tools, and the increased chance of security breaches[4,5,6,7,8]
- the level of consent required[8]
- the blurred limits of duty of care, and the challenge of defining and maintaining boundaries with clients, when their access to a provider may be effectively unlimited[8,9]
- the absence of clearly defined guidelines in navigating privacy, security and confidentiality.

Emergency and crisis situations

- Interviews with psychologists revealed that 52% felt they had inadequate skills to manage an emergency or crisis situation in the context of online counselling.[6]
- Providers note the difficulty in identifying a client's mental status and changes in status, and in addressing these changes, when using a digital tool.[6,9]
- Online counselling through video conferencing, and particularly group discussion, may exacerbate stress for some clients. How best to identify and address individuals' needs in a virtual group-based format is a concern.[10]
- Mental health care providers worry about how to manage crisis information that may be conveyed through digital mental health tools.[11]

Face-to-face contact

Research shows that mental health care providers are beginning to incorporate digital health tools in providing ongoing face-to-face services.[6] They express strong interest in the ability of these tools to extend therapy beyond each individual session.[4] However, there are also concerns about this trend:

- One study noted that clients' potential lack of support outside of the therapy room could be exacerbated by sole reliance on digital mental health interventions.[4]
- Clinical practice relies on non-verbal cues communicated in person to develop the therapeutic alliance, so technology-based interactions may sometimes not be as effective.[9]
- Face-to-face contact can provide a valuable avenue for some clients to separate from technology and develop the social skills needed for interpersonal communication and connection[9]—so this kind of tool may not be indicated in all cases.

Reliability of digital tools and technologies

Providers may need to discuss the reliability of digital tools and technologies with clients. This may include discussing the challenges in choosing the most appropriate tool, especially for clients with complex and comorbid clinical presentations, or presentations that change over time.[2,7,11,12] It is possible that unintended consequences may arise from using digital mental health tools that lack empirical support.[7]

Not all providers may be open to using technology-based interventions, leading to additional skepticism about reliability.[2,11]

Logistical factors

Three logistical factors to consider are training, time, and the interaction between new tools and existing policy and procedures:

- Barriers reported in the literature include inadequate training for staff on how to work with and navigate digital mental health tools.[2,6,9]
- The time needed to learn some of the tools may also be a barrier,[2,9,11] particularly given the full caseloads of many mental health care providers.
- In implementing a tool, the impact on billing, malpractice insurance and coverage, liability, licensure and cost need to be addressed.[6,7,11]

Additional considerations

- Digital health interventions can perpetuate the "digital divide," whereby not all clients have the access or the technological skills needed to engage in digital mental health services.[11,13] In these cases, health care providers can support clients in obtaining access to technology, if appropriate, or in seeking support through a different source.
- There is the potential in some people for overreliance on technology. For example, one study commented on the possibility of technology use becoming a maladaptive safety behaviour for some clients, increasing distress when access to the technology is unavailable, and thus creating dependence rather than promoting autonomy.[8]
- Language may be a barrier to technology use in cases where the client is using a tool in a language they are not comfortable with.[3]

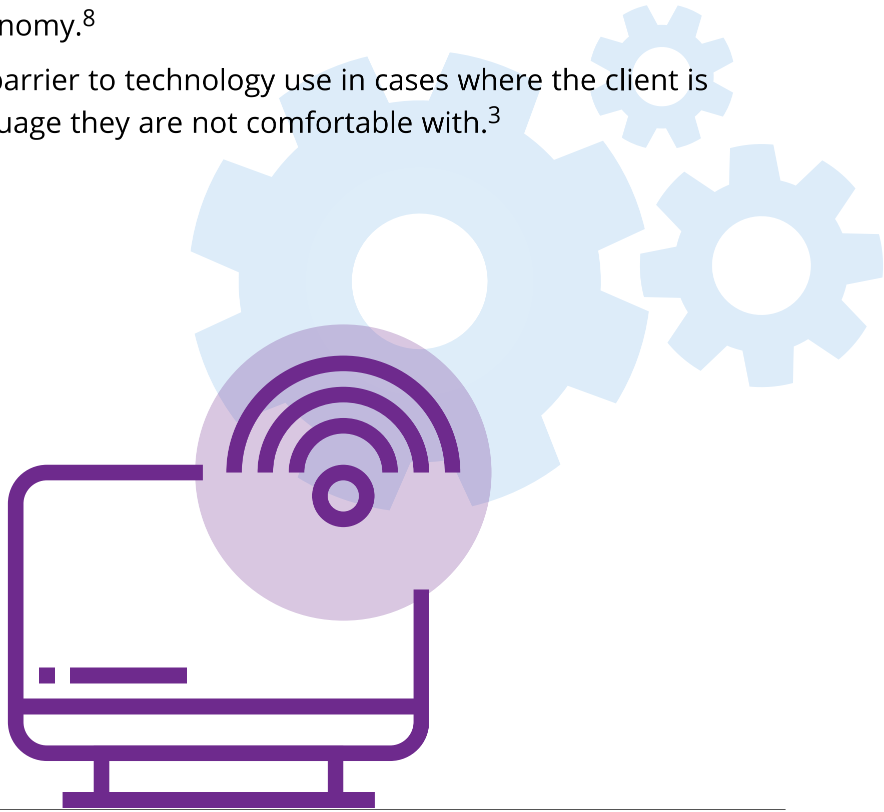

Is this the right tool for you? Questions for clients

Many clients are interested in digital mental health tools and may research them on their own, while others may come to a provider seeking information on tools. Research has identified many factors that can help a client use a digital tool successfully, as well as factors that may impede success.

This section highlights factors that may respectively help or hinder clients' use of digital mental health tools. It presents a series of questions, compiled through a comprehensive literature review, on the barriers and facilitators of digital mental tool uptake among clients.* You can help a client understand what might help them use a tool consistently by asking appropriate questions from the lists below, which correspond to different types of tool. (However, the questions will not recommend a specific tool, or how best to use a tool with your client.)

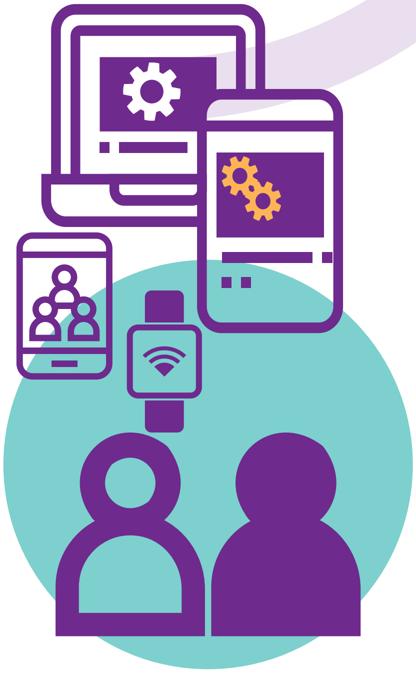

* These questions are based on a literature review to identify the facilitators of, and barriers to, client uptake of digital mental health tools, completed by Hwayeon Danielle Shin, RN, Dalhousie University. The categories of technology type are adapted from the Mental Health Commission of Canada.

General questions about digital health tools

Questions to identify potential success factors or areas of concern

- Are you interested in using digital health tools in general?
- Do you know where to find the kind of tool you're looking for?
- Does the tool have features you want to use?
- Are you confident the tool is accurate, effective and trustworthy?
- Does the tool seem user-friendly? For example, is it easy to navigate? Can you understand what to do next?
- How much does it cost to use the tool? Is there a one-time cost, or do you need to keep paying?
- Does the tool have clear privacy and security policies?
- Is it clear what personal data is being collected and why? How much personal data are you comfortable sharing?
- Can you find more information on the tool as you learn to use it?
- Are you comfortable with the language used in the tool?

Computerized interventions, resources and apps

Questions to identify potential success factors

- Does the tool seem convenient and flexible, so you can adapt it to your life?

Questions to identify potential areas of concern

- Would any personal reasons, like your health, or how much time you have, make it difficult to use the tool?
- Is there any part of the design or function of the tool that makes it unusable for you? For example, does it have the right level of security, come with enough instructions, send an appropriate number of alerts?
- Many apps are not for emergency use; would this be a problem for you?

Wearable computing and monitoring devices

Questions to identify potential success factors

- Do you think you can use the information from the device to help yourself? How might you use it?
- Do you know other people who use this tool?

Questions to identify potential areas of concern

- Are there any practical issues in your life that might make the tool inconvenient?
- Think about design features that will be important to you. For example, does the tool need to have a long battery life? Are the buttons easy to find and press? Is the screen text big enough?
- Do you need help remembering to use the tool? Does the tool have a reminder function?

Peer support through social media and other technologies

Questions to identify potential success factors

- Do you find support in social connections?
- Do you like to share stories, information and coping strategies, and learn from others?
- Do you value the support of your peers and the ability to give and take advice?
- Have other people suggested that you join an online community?
- Would it be an advantage to you to receive online support instead of face-to-face support?
- Do you want to have access to moderators or other professional support through the tool?

Questions to identify potential areas of concern

- Do you think you might have emotions come up or feel vulnerable while online? What could help with this?
- Are you concerned about getting overloaded with information from online communities? Do you think you might get misleading information?
- Is it possible using an online community may make you more worried about your health condition?

Gaming

Questions to identify potential success factors

- Does the tool seem to be entertaining?
- Does the tool look like it would hold your attention?
- Would you consider it an advantage if the tool provided access to therapy guidance?

Questions to identify potential areas of concern

- Do you think the game might distract you from therapy?
- Do you think it might lead you to connect less with other people in real life?
- Do you think you may not be able to stop playing the game?
- Do you think the game might lead you into its world and away from reality?
- Do you wonder if the game is appropriate for you as part of your care?
- Do you think the game might make your symptoms worse?

Telemedicine and telehealth

Questions to identify potential success factors

- Is the convenience and flexibility offered by the tool an advantage to you?
- If using the tool meant no wait time to receive care, would you like it more?
- Is the fact that the tool offers you access to a “live” person an advantage to you?
- If the tool offered a way to “get and give” support to other clients like you, would that be of interest to you?

Questions to identify potential areas of concern

- Can you think of any practical issues in your life that might make the tool inconvenient?
- Would any personal reasons, like your health or how much time you have, make it difficult to use the tool?
- Do you prefer in-person visits over video visits?

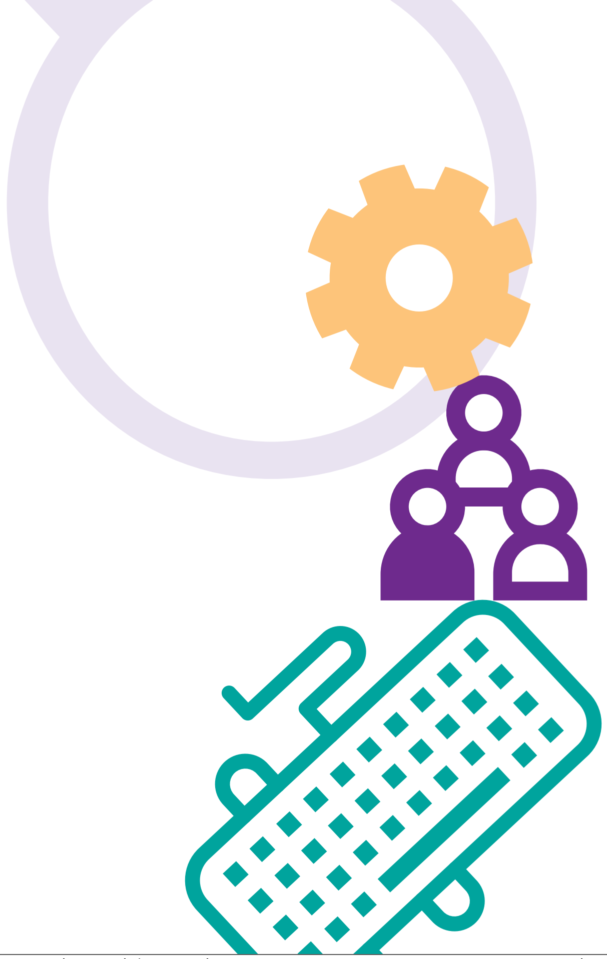

Resource chart

	Resource	Audience	Format	Specific to mental health?	Languages	Country of origin
**App rating resources**	Psyberguide	CC, P	Web	Y	E	US
	Addiction and Mental Health Mobile Application Directory 2019	P	PDF	Y	E	CAN
	Practical Apps	P	Web	N	E, F	CAN
**App assessment guidelines or frameworks**	Mental Health Apps: How to Make an Informed Choice	P, CC	PDF	Y	E, F	CAN
	HITEQ Health App Decision Tree	P	PDF	N	E	US
	Checklist: Five Things to Think About While Assessing E-Mental Health Tools	P	PDF	Y	E, F	CAN
	App Evaluation Model (APA)	P	Web	Y	E	US
**Implementation resources**	Toolkit for E-Mental Health Implementation	P	PDF	Y	E, F	CAN
	E-Mental Health: A Guide for GPs	P	PDF	Y	E	AUS
	Texting for Better Care Toolkit	P	Web	N	E	US
	Using Digital Mental Health Tools to Enhance Your Practice	P	PDF	Y	E	AUS
**Resources to improve communication**	Ask Me about Digital	P, CC	PDF	N	E	HUN
	Computers in the Clinic comics	CC, P	PDF	N	E	US
	Social Media: Practical Guidance and Best Practice	P	Web	N	E	UK
	Strategies for Engaging Patients in E-Mental Health	P	PDF	Y	E, F	CAN
**Resources specifically for clients**	Mental Health, Technology and You	CC	PDF	Y	E, F	CAN
	How to Protect Yourself Online	CC	PDF	Y	E, F	CAN
	What's Your Journey?	CC	PDF	Y	E, F	CAN

Legend

CC: Clients and caregivers
P: Providers

AUS: Australia
CAN: Canada
HUN: Hungary
UK: United Kingdom
US: United States

Additional resources

App libraries

- App Library (Health Navigator New Zealand)

  https://www.healthnavigator.org.nz/apps/

- Apps Library (National Health Service, United Kingdom)

  https://www.nhs.uk/apps-library/

App frameworks

- Guiding Principles for Physicians Recommending Mobile Health Applications (Canadian Medical Association)

  https://policybase.cma.ca/en/permalink/policy11521

Implementation guides

- Digital Health Implementation Playbook (American Medical Association)

  https://www.ama-assn.org/amaone/ama-digital-health-implementation-playbook

Therapy decision guides

- Therapy option guide (Anxiety and Depression Association of America)

  https://adaa.org/sites/default/files/final-therapygu_23840851_3ad732e6e2a37020f3ac49fcaf48f6305f631dcf_0.jpeg

Comprehensive web resources

- E-Mental Health in Practice (Black Dog Institute, Australia)

  https://www.blackdoginstitute.org.au/education-training/health-professionals/ementalhealth-in-practice

- Improving Patient-Centred Technology Use (iPaCT) Education and Evaluation Toolkit (University of Chicago)

  https://www.mededportal.org/publication/9953/

Resource summaries

Psyberguide

**Type**	App rating website
**Creator**	One Mind, in partnership with University of California, Irvine, and Northwestern University
**Country**	United States
**URL**	www.psyberguide.org/apps
**Date**	No date provided, but appears to be updated regularly
**Audience**	Clients; also appropriate for providers, caregivers
**Format**	Website
**Languages**	English
**Description**	An extensive collection of mental health apps reviewed by experts and rated according to three parameters: credibility, user experience and transparency. Allows for searching by condition or treatment type, and filtering by score, platform availability, audience and cost. Also tracks apps and reviews that are no longer available.
**Requires internet connection to use?**	Yes
**Includes practical examples of use?**	No
**Applicable to Canada?**	Yes, though not clear how many apps are available in Canada
**Collects data on user?**	Website uses cookies<br>Apps reviewed on site will vary in the amount of data collected
**Specific to mental health?**	Yes<br>Can be searched by condition
**Who would find it useful?**	Anyone looking to choose a mental health app, including clients and providers
**Designed for use in client interaction?**	Can be used during a visit to research potential apps<br>Can be used by client or provider alone to research apps

Addiction and Mental Health Mobile Application Directory 2019

**Type**	App directory
**Creator**	Alberta Health Services, and Alberta Addiction and Mental Health Research Partnership Program
**Country**	Canada
**URL**	https://www.albertahealthservices.ca/assets/info/res/mhr/if-res-mhr-kt-mobile-app-directory.pdf
**Date**	2019; updated annually
**Audience**	Providers; secondarily researchers and app developers
**Format**	PDF
**Languages**	English
**Description**	A static directory of mental health apps by condition, with a summary of features plus links to any available research studies or expert reviews. 100+ apps in 2019 edition. Inclusion criteria are applied in compiling directory, but otherwise individual apps are not reviewed or rated.
**Applicable to Canada?**	Yes; developed in Canada by Alberta provincial government
**Requires internet connection to use?**	Once downloaded, PDF document can be used without internet connection; however, internet connection required to access external hyperlinks within document
**Includes practical examples of use?**	No
**Collects data on user?**	No<br>Apps reviewed in document will vary in the amount of data collected
**Specific to mental health?**	Yes<br>Apps categorized by condition
**Who would find it useful?**	Providers or administrators looking for information on digital mental health apps<br>Providers looking for digital mental health apps to supplement care
**Designed for use in client interaction?**	No; uses technical language and does not provide a review or rating

Practical Apps

**Type**	App rating website
**Creator**	Ontario Telemedicine Network
**Country**	Canada
**URL**	https://practicalapps.ca/
**Date**	Oct. 2016 – Mar. 2019; each review collection clearly dated
**Audience**	Primary care providers
**Format**	Website
**Languages**	English, French
**Description**	Physician-led reviews of collections of apps, organized by health condition (mental health and broader). Reviews include summary of condition and role of digital health tools, detailed review using 6 parameters, and expert or patient experience perspectives (of the condition, not the digital health tool)
**Applicable to Canada?**	Yes; developed in Canada by Ontario provincial government
**Requires internet connection to use?**	Yes
**Includes practical examples of use?**	No
**Collects data on user?**	Website uses cookies
**Specific to mental health?**	No<br>6 mental health conditions addressed: PTSD, adult anxiety, child and adolescent anxiety, alcohol consumption, insomnia, smoking Total of 23 apps reviewed
**Who would find it useful?**	Providers looking for basic knowledge of specific mental healthcondition and detailed review of a limited number of apps available in Canada
**Designed for use in client interaction?**	No

Mental Health Apps: How to Make an Informed Choice

**Type**	App assessment resource
**Creator**	Mental Health Commission of Canada
**Country**	Canada
**URL**	https://www.mentalhealthcommission.ca/sites/default/files/2018-01/eMH_app_eng.pdf<br>https://mhealth.jmir.org/2018/7/e10016/ (research article supporting tool)
**Date**	2018
**Audience**	Providers (from peer support workers to social workers, nurses and psychologists), and clients and caregivers
**Format**	PDF
**Languages**	English, French
**Description**	Guiding principles and selection criteria for choosing an app
**Applicable to Canada?**	Yes, developed by Canadian organization with national mandate
**Requires internet connection to use?**	Once downloaded, PDF document can be used without internet connection
**Includes practical examples of use?**	No
**Collects data on user?**	No
**Specific to mental health?**	Yes
**Who would find it useful?**	Providers looking for general guidance on how to evaluate a digital mental health tool
**Designed for use in client interaction?**	Not primarily<br>Could be shared with informed clients as part of a general discussion on principles for assessing an app

HITEQ Health App Decision Tree

**Type**	App assessment resource
**Creator**	Health IT, Evaluation and Quality Center (funded by US Department of Health and Human Services)
**Country**	United States
**URL**	https://hiteqcenter.org/Resources/Electronic-Patient-Engagement/Mobile-Health/hiteq-health-app-decision-tree
**Date**	Not on document; linked to a blog post from Sept. 2017
**Audience**	Providers
**Format**	PDF
**Languages**	English
**Description**	A visual decision algorithm that poses a series of questions about different characteristics of an app, in order to determine its suitability
**Applicable to Canada?**	Yes; American, but the information provided also applies in the Canadian context
**Requires internet connection to use?**	Once downloaded, PDF document can be used without internet connection
**Includes practical examples of use?**	No
**Collects data on user?**	No
**Specific to mental health?**	No; general to health apps
**Who would find it useful?**	Someone looking for a step-by-step process for assessing a health app
**Designed for use in client interaction?**	No, language and content is specific to providers

Checklist: Five Things to Think About While Assessing E-Mental Health Tools

**Type**	Part of toolkit, p. 24
**Creator**	Mental Health Commission of Canada
**Country**	Canada
**URL**	https://www.mentalhealthcommission.ca/English/e-mental-health
**Date**	2018
**Audience**	Providers
**Format**	PDF
**Languages**	English, French
**Description**	A checklist of questions to ask when evaluating any e-mental health tool
**Applicable to Canada?**	Yes, deveoped by Canadian organization with national mandate
**Requires internet connection to use?**	Once downloaded, PDF document can be used without internet connection
**Includes practical examples of use?**	No
**Collects data on user?**	No
**Specific to mental health?**	Yes
**Who would find it useful?**	Any care provider assessing a digital mental health tool
**Designed for use in client interaction?**	No

App Evaluation Model

**Type**	App assessment model
**Creator**	American Psychiatric Association
**Country**	United States
**URL**	https://www.psychiatry.org/psychiatrists/practice/mental-health-apps/app-evaluation-model
**Date**	Not stated
**Audience**	Providers, specifically psychiatrists
**Format**	Website
**Languages**	English
**Description**	A hierarchical 5-step model that allows providers to evaluate an app before using it in their practice. The evaluation topics are: background info; risk/privacy and security; evidence; ease of use; interoperability
**Applicable to Canada?**	Yes; American, but the information provided also applies in the Canadian context
**Requires internet connection to use?**	Yes
**Includes practical examples of use?**	Yes
**Collects data on user?**	Website uses cookies
**Specific to mental health?**	Yes
**Who would find it useful?**	Psychiatrists looking for guidance on how to perform their own evaluation of an app
**Designed for use in client interaction?**	No

Toolkit for E-Mental Health Implementation

**Type**	Implementation guide
**Creator**	Mental Health Commission of Canada
**Country**	Canada
**URL**	https://www.mentalhealthcommission.ca/English/e-mental-health
**Date**	2018
**Audience**	Providers (from peer support workers to social workers, nurses and psychologists)
**Format**	PDF
**Languages**	English, French
**Description**	A set of strategies to plan and implement e-mental health programs in clinical practice. Modules are: i) exploring the world of e-mental health; ii) launching and sustaining uptake; iii) building your digital skill set; iv) engaging clients in e-mental health; and v) leadership for e-mental health innovation. Provides extensive information on increasing capacity around digital mental health tools, incorporating them into practice, using them with clients and providing leadership in innovation. Contains case studies and many worksheets.
**Applicable to Canada?**	Yes; developed by Canadian organization with national mandate
**Requires internet connection to use?**	Once downloaded, PDF document can be used without internet connection
**Includes practical examples of use?**	Yes
**Collects data on user?**	No
**Specific to mental health?**	Yes
**Who would find it useful?**	Providers looking for a range of information, from guidance on full implementation projects to simpler practical tips on working with clients to take up digital mental health tools
**Designed for use in client interaction?**	No<br>Accompanying client guide, *Mental Health, Technology and You*, is designed for clients

E-Mental Health: A Guide for GPs

**Type**	Implementation guide
**Creator**	Royal Australian College of General Practitioners
**Country**	Australia
**URL**	https://www.racgp.org.au/FSDEDEV/media/documents/Clinical%20Resources/Guidelines/Mental%20health/e-mentalhealth-guide.pdf
**Date**	Published 2015, updated 2018
**Audience**	Providers, specifically family physicians
**Format**	PDF
**Languages**	English
**Description**	Guide based on practical questions of using e-mental health tools in practice. Sections include: i) description, benefits, choosing clients; ii) exploring the world of e-mental health; iii) deciding how to use e-mental health; iv) talking to clients; and v) managing clients who are using e-mental health
**Applicable to Canada?**	Concepts are applicable; however, all links to resources are Australian
**Requires internet connection to use?**	Once downloaded, PDF document can be used without internet connection
**Includes practical examples of use?**	Yes
**Collects data on user?**	No
**Specific to mental health?**	Yes, and targets non–mental health providers
**Who would find it useful?**	Care providers, particularly family physicians, who are not experts in mental health but care for clients
**Designed for use in client interaction?**	No

Texting for Better Care Toolkit

**Type**	Implementation guide
**Creator**	Center for Care Innovation
**Country**	United States
**URL**	https://www.careinnovations.org/resources/texting-better-care-toolkit/
**Date**	Nov. 2017
**Audience**	Providers
**Format**	Website
**Languages**	English
**Description**	Basic toolkit on how to implement a texting program in a clinic; topics are: i) selecting a vendor; ii) developing messaging content and logic; iii) obtaining consent and ensuring privacy; iv) implementation use case; v) external resources; vi) research on texting
**Applicable to Canada?**	Generally, yes. All examples are from US, includes references to US privacy law, etc., but general ideas are transferable
**Requires internet connection to use?**	Yes
**Includes practical examples of use?**	Yes
**Collects data on user?**	Website uses cookies
**Specific to mental health?**	No
**Who would find it useful?**	Administrators developing a texting program
**Designed for use in client interaction?**	No

Using Digital Mental Health Tools to Enhance Your Practice

**Type**	Podcast
**Creator**	Black Dog Institute
**Country**	Australia
**URL**	https://www.blackdoginstitute.org.au/clinical-resources/health-professional-resources/podcasts-for-health-professionals/being-well-podcast-series<br>Scroll to find "Using Digital Mental Health Tools to Enhance Your Practice" parts 1 and 2
**Date**	2019
**Audience**	Providers
**Format**	Podcast (via website)
**Languages**	English
**Description**	Two podcasts on how to select and evaluate digital mental health tools, and how to blend tools into therapy
**Applicable to Canada?**	Australia-specific; however most information is generic enough to apply to Canada
**Requires internet connection to use?**	Yes
**Includes practical examples of use?**	Yes
**Collects data on user?**	Website uses cookies
**Specific to mental health?**	Yes
**Who would find it useful?**	Providers looking for an introduction to choosing and integrating mental health tools into practice
**Designed for use in client interaction?**	No; podcasts are specific to providers

Ask Me about Digital

**Type**	Awareness campaign
**Creator**	The Medical Futurist Institute
**Country**	Hungary
**URL**	https://medicalfuturist.com/guide-patient-questions-digital-health/
**Date**	2018
**Audience**	Providers, clients, caregivers
**Format**	Website and PDF
**Languages**	English
**Description**	At its core, a badge/pin for providers that indicates their openness to talking about digital health technologies and digital communication. It is meant to be a conversation starter. The accompanying guide is an FAQ for providers about evaluating digital tools, answering patients' questions and using digital communication technologies in patient care.
**Applicable to Canada?**	Is US-focused, but much of the information is relevant to Canada
**Requires internet connection to use?**	Once downloaded, PDF document can be used without internet connection
**Includes practical examples of use?**	Yes; has an associated group on LinkedIn
**Collects data on user?**	Website uses cookies
**Specific to mental health?**	No
**Who would find it useful?**	Any care provider interested in starting a conversation about digital health tools
**Designed for use in client interaction?**	Yes

Computers in the Clinic comics

**Type**	Key message posters
**Creator**	Alkureishi et al. (University of Chicago) and Gold Foundation
**Country**	United States
**URL**	Client version: https://s3.amazonaws.com/gold-foundation/wp-content/uploads/2018/01/Alkureishi-Czerwiec-Arora-Lee_patient_comic.pdf<br>Provider version: https://s3.amazonaws.com/gold-foundation/wp-content/uploads/2019/06/Alkureishi-Czerwiec-Arora-Lee_provider_comic.pdf
**Date**	2016
**Audience**	One version for clients, one for providers
**Format**	PDF
**Languages**	English
**Description**	Two comic-based posters highlighting key messages about use of technology in the patient-provider interaction. One targets the patient and how they can work with their doctor and technology. The other provides messages to the provider on how to use technology with their patient.
**Applicable to Canada?**	Yes; content is generic and applicable to Canada
**Requires internet connection to use?**	Once downloaded, PDF document can be used without internet connection
**Includes practical examples of use?**	No
**Collects data on user?**	No
**Specific to mental health?**	No
**Who would find it useful?**	Any care provider who uses a computer while seeing a client
**Designed for use in client interaction?**	Yes

Social Media: Practical Guidance and Best Practice

**Type**	Guidance document
**Creator**	British Medical Association
**Country**	United Kingdom
**URL**	https://www.bma.org.uk/-/media/files/pdfs/employment advice... https://www.bma.org.uk/advice/employment/ethics/social-media-guidance-for-doctors
**Date**	2018
**Audience**	Providers
**Format**	PDF
**Languages**	English
**Description**	A web page and guidance document introducing social media to providers, giving examples of uses, and presenting issues of privacy and harassment
**Applicable to Canada?**	Yes; most information is generic and applicable to Canada; links for support or further reading are specific to the UK
**Requires internet connection to use?**	Yes
**Includes practical examples of use?**	No
**Collects data on user?**	Website uses cookies
**Specific to mental health?**	No
**Who would find it useful?**	Any care provider contemplating using social media
**Designed for use in client interaction?**	No

Strategies for Engaging Clients in E-Mental Health

**Type**	Part of toolkit (pp. 67–69)
**Creator**	Mental Health Commission of Canada
**Country**	Canada
**URL**	https://www.mentalhealthcommission.ca/English/e-mental-health
**Date**	2018
**Audience**	Providers
**Format**	PDF
**Languages**	English, French
**Description**	A stage-wise description of strategies for engaging patients in e-mental health initiatives; provides practical steps
**Applicable to Canada?**	Yes, developed by Canadian organization with national mandate
**Requires internet connection to use?**	Once downloaded, PDF document can be used without internet connection
**Includes practical examples of use?**	Yes
**Collects data on user?**	No
**Specific to mental health?**	Yes
**Who would find it useful?**	A care provider looking for information specific to engaging clients with around digital mental health tools
**Designed for use in client interaction?**	No

Mental Health, Technology and You

**Type**	Client guide
**Creator**	Mental Health Commission of Canada
**Country**	Canada
**URL**	https://www.mentalhealthcommission.ca/English/media/3933
**Date**	2018
**Audience**	Clients, caregivers
**Format**	PDF
**Languages**	English, French
**Description**	Client guide with descriptions of technologies, a journey map, personal stories and online safety tips. Designed as an introduction to digital mental health tools
**Applicable to Canada?**	Yes; developed by Canadian organization with national mandate
**Requires internet connection to use?**	Once downloaded, PDF document can be used without internet connection
**Includes practical examples of use?**	Includes personal stories of people using digital mental health tools
**Collects data on user?**	No
**Specific to mental health?**	Yes
**Who would find it useful?**	Any client or care provider looking for a client guide introducing different digital mental health tools
**Designed for use in client interaction?**	Yes, and can be used by client alone

How to Protect Yourself Online

**Type**	Part of client guide (p. 12)
**Creator**	Mental Health Commission of Canada
**Country**	Canada
**URL**	https://www.mentalhealthcommission.ca/English/media/3933
**Date**	2018
**Audience**	Clients, caregivers
**Format**	PDF
**Languages**	English, French
**Description**	Practical tips for staying safe online
**Applicable to Canada?**	Yes; developed by Canadian organization with national mandate
**Requires internet connection to use?**	Once downloaded, PDF document can be used without internet connection
**Includes practical examples of use?**	No
**Collects data on user?**	No
**Specific to mental health?**	Yes
**Who would find it useful?**	Client or provider looking for information about privacy and security online
**Designed for use in client interaction?**	Yes, and can be used by client alone

What's Your Journey?

**Type**	Part of client guide (p. 11)
**Creator**	Mental Health Commission of Canada
**Country**	Canada
**URL**	https://www.mentalhealthcommission.ca/English/media/3933
**Date**	2018
**Audience**	Clients, caregivers
**Format**	PDF
**Languages**	English, French
**Description**	A graphic depiction of different digital mental health tools and their use in mental health care
**Applicable to Canada?**	Yes; developed by Canadian organization with national mandate
**Requires internet connection to use?**	Once downloaded, PDF document can be used without internet connection
**Includes practical examples of use?**	No
**Collects data on user?**	No
**Specific to mental health?**	No
**Who would find it useful?**	Client or provider looking for overview of different digital mental health tools
**Designed for use in client interaction?**	Yes, and can be used by client alone

Project summary

Project genesis

In Canada, numerous digital health tools have been developed specifically for people with mental illness, which has the potential to lead to significant benefits. However, engagement of providers and clients with these tools is often inconsistent, and in many cases poor. Uptake needs to be improved for benefits to be realized. Some resources to support the uptake of these digital tools have been developed, but these resources are not well known and have not been consolidated into a single document. Thus, a document that aims to consolidate these resources was identified as a need.

Guiding principles

Discussions by the project team, along with valuable feedback from stakeholders from across Canada—including those with lived experience of mental illness—led to the definition of guiding principles for the project. These principles include the following:

- The client is the focus of the interaction, even though the audience for this document is health care providers.
- Empowerment is crucial for behaviour change, as acknowledged in several behaviour change models, such as the theory of planned behaviour and the health belief model.[14,15]
- Through support and choice, the client is empowered to use e-mental health tools in the way they deem most appropriate.
- Empowering health care providers to feel more comfortable with technology may increase their self-perceived competence with their clients.
- Practicality and user-friendliness are key objectives of the design of the document.
- We will define clearly the audience of the project, including who is not the audience.
- We will acknowledge the lack of tools that target Indigenous people.

Project design

The document was developed using methods recommended by the United States Agency for Healthcare Research and Quality (AHRQ) on toolkit creation.[16]

A focused literature review and environmental scan was completed in autumn 2019. The search methodology is outlined below. Search results were assessed using criteria determined by team discussion. Preference was given to resources that were directly applicable to a provider-client interaction in the Canadian setting, that contained no or little jurisdiction-dependent information, that were relatively recent and that were available in both English and French.

In early January 2020, a half-day workshop was convened to elicit stakeholders' feedback on a draft document content outline and draft resource summary, and to garner information on dissemination techniques. Among the 16 workshop participants were client advocates, health care providers, policy makers and academics. Two activities—a structured brainstorming activity and small group discussions—were used to elicit feedback on the drafts. Notes were recorded by the project team and then compiled and summarized to inform the final draft of the document. The project team maintained a list of workshop outcomes that were addressed in the revisions. This information was shared with workshop participants. The structure of the final document was informed by input from the stakeholder workshop.

Resource identification and search methodology

Environmental scan

We began by compiling a list of experts in digital mental health from across Canada, and contacted them requesting information on any known resources related to the project. The list was compiled from professional contacts of the study team, known experts in digital mental health in Canada, and experts who had participated in similar projects. Experts also recommended others who should be contacted.

The list of experts who contributed their knowledge and expertise is listed in Appendix 2. In total, 31 experts plus 4 project team members were interviewed. Geographic representation was as follows: 16 from Atlantic Canada, 2 from Quebec, 13 from Ontario, 1 from the Prairies, 1 from British Columbia and 2 from the United States. Experts included health care providers, academics,

industry experts and program directors. Client perspectives were included during the stakeholder workshop.

Grey literature search

An online search in September and October 2019, using the Google search engine, specific search terms and a targeted organizational website search, resulted in the review of 91 sites in total.

The following searches were undertaken, and the first 10 pages (minimum) were reviewed for potential results:

- (electronic OR digital OR mobile) AND "mental health" AND (tool OR resource OR e-tool OR e-resource OR toolkit OR app OR web)
- (electronic OR digital OR mobile) AND patient AND (tool OR resource OR e-tool OR e-resource OR toolkit OR app OR web)—first 20 pages were reviewed
- digital mental health tool
- digital tools to help my mental health.

Additionally, the websites of specific organizations, listed in Appendix 3, were also reviewed for applicable resources.

Literature reviews and gaps analysis

Two literature scans were completed by student members of the project team. The first review, completed by Vanessa Strong, identified literature on facilitators and barriers to the engagement of mental health providers in digital health tools. The second review, completed by Hwayeon Danielle Shin, identified literature on facilitators and barriers to mental health clients' engagement in digital health tools. Details of the search strategies for each review are available on request.

These reviews were synthesized and incorporated into this document based on feedback from the stakeholder workshop. Analysis of the collection was completed by student team member Alanna Miller. Summary statistics on the resources were tabulated. The Mental Health Commission of Canada's digital tools typology was used as a framework for a gaps analysis of the current collection of resources.[1] Pertinent results are included in the introduction of this document.

REFERENCES

1. Mental Health Commission of Canada. E-mental health in Canada: Transforming the mental health system using technology [Internet]. Ottawa, ON: MHCC. 2014 [cited 2019 Sep]. Available from https://www.mentalhealthcommission.ca/sites/default/files/MHCC_E-mental_Health-Briefing_Document_ENG_0.pdf

2. Clarke AM, Chambers D, Barry MM. Bridging the digital disconnect: Exploring the views of professionals on using technology to promote young people's mental health. Sch Psychol Int. 2017;38:380–97.

3. Ferrari M, Ahmad F, Shakya Y, Ledwos C, McKenzie K. Computer-assisted client assessment survey for mental health: Patient and health provider perspectives. BMC Health Serv Res. 2016;16:516.

4. Schueller SM, Washburn JJ, Price M. Exploring mental health providers' interest in using web and mobile-based tools in their practices. Internet Interv. 2016;4:145–51.

5. Eonta AM, Christon L, Hourigan S, Ravindran N, Vrana S, Southam-Gerow M. Using everyday technology to enhance evidence-based treatments. Prof Psychol Res Pract. 2011;42:513–20.

6. Glueckauf RL, Mahue MM, Drude KP, Wells BA, Wang Y, Gustafson DJ, et al. Survey of psychologists' telebehavioral health practices: Technology use, ethical issues, and training needs. Prof Psychol Res Pract. 2018;49:205–19.

7. Hatch A, Hoffman JE, Ross R, Docherty JP. Expert consensus survey on digital health tools for patients with serious mental illness: Optimizing for user characteristics and user support. JMIR Ment Health. 2018;5:e46.

8. Ignatowicz A, Slowther AM, Elder P, Bryce C, Hamilton K, Huxley C, et al. Ethical implications of digital communication for the patient-clinician relationship: Analysis of interviews with clinicians and young adults with long term conditions (the LYNC study). BMC Med Ethics. 2018;19:11.

9. Orlowski S, Lawn S, Matthews, B, Venning A, Wyld K, Jones G, et al. The promise and the reality: A mental health workforce perspective on technology-enhanced youth mental health service delivery. BMC Health Serv Res. 2016;16:562.

10. Sansom-Daly UM, Wakefield CE, McGill BC, Patterson P. Ethical and clinical challenges delivering group-based cognitive behavioural therapy to adolescents and young adults with cancer using videoconferencing technology. Aust Psychol. 2015;50:271–8.

11. Lattie EG, Nicholas J, Knapp AA, Skerl JJ, Kaiser SM, Mohr DC. Opportunities for and tensions surrounding the use of technology-enabled mental health services in community mental health care. Adm Policy Ment Health. 2020;47:139–49.

12. Berry N, Bucci S, Lobban F. Use of the internet and mobile phones for self-management of severe mental health problems: Qualitative study of staff views. JMIR Ment Health. 2017;4:e52.

13. Bucci S, Schwannauer M, Berry N. The digital revolution and its impact on mental health care. Psychol Psychother Theory Res Pract. 2019;92:277–97.

14. Ajzen I. The theory of planned behavior. Organ Behav Hum Decis Process. 1991;50:179–211.

15. Rosenstock IM. Historical origins of the health belief model. Health Educ Monograph. 1974;2:328–35.

16. Agency for Healthcare Research and Quality. AHRQ publishing and communications guidelines. section 1: product development. Toolkit [Internet]. Rockville, MD: AHRQ. Created 2013 Feb. Reviewed 2020 Jan [cited 2019 May]. Available from www.ahrq.gov/research/publications/pubcomguide/pcguide1a.html##_Toc7696924

APPENDIX 1: GLOSSARY

Administrator: A person working in the mental health care sector who performs business operations roles that support the clinical care functions of an organization.

App assessment resource: A resource that provides a generic model or framework that the user follows to evaluate an app of their choice; the resource does not provide an assessment of an app but rather guidance on how to assess an app.

App rating resource: A resource that provides summaries and expert ratings of different apps; the resource offers an expert assessment of different apps.

Caregiver: An informal care provider, such as a family member, friend or other social support, whom a client calls upon for help, care and support.

Client: A person who accesses mental health services or seeks mental health care.

Health care provider: A trained health professional or allied health worker who provides care and services to people looking for mental health care.

Implementation resource: A resource that provides information and guidance on how to establish and operate a program or practice that incorporates a digital mental health tool; these resources often target larger projects that involve changes to workflow and care practices.

Resource: A website, document, poster, or other material that can help to integrate different digital mental health tools into practice; for example, a website that reviews and rates apps can help identify the right tool for a client.

Tool: An app, website, device or other digital mental health technology that is to be used by the client or the provider, together or separately; for example, a tool could be a cognitive behavioural therapy app.

APPENDIX 2: ENVIRONMENTAL SCAN PARTICIPANTS

Contact	Province	Organization
Kelly Anderson	Ontario	Western University
Alexa Bagnell	Nova Scotia	IWK Health Centre
Krista Balenko	Quebec	Canada Health Infoway
Kim Barro	Nova Scotia	Nova Scotia Department of Health and Wellness
Richard Booth	Ontario	Western University
Elizabeth Cawley	Newfoundland and Labrador	Association of Atlantic Universities
AnnMarie Churchill	Newfoundland and Labrador	Memorial University of Newfoundland
Peter Cornish	Newfoundland and Labrador	Memorial University of Newfoundland
Cheryl Forchuk	Ontario	Western University
David Gratzer	Ontario	Centre for Addiction and Mental Health
Heather Hadjistavropoulous	Saskatchewan	University of Regina
Jennifer Heatley	Nova Scotia	Nova Scotia Department of Health and Wellness
Amanda Hudson	Prince Edward Island	Health PEI
Danielle Impey	Ontario	Mental Health Commission of Canada
Sean Kidd	Ontario	Centre for Addiction and Mental Health
Anne Kirvan	Ontario	Centre for Addiction and Mental Health
Karim Keshavjee	Ontario	InfoClin
Stan Kutcher	Nova Scotia	Dalhousie University
Ashwin Kutty	Nova Scotia	We Us Them
Shalini Lal	Quebec	Université de Montréal
Patricia Lingley-Pottie	Nova Scotia	Dalhousie University
Stacy Lloyd	United States	American Medical Association
Rita MacAulay	Nova Scotia	Nova Scotia Department of Health and Wellness
Gisele Maillet	New Brunswick	New Brunswick Department of Health
Matt Menning	United States	American Medical Association
Erin Michalak	British Columbia	University of British Columbia
Josh Rash	Newfoundland and Labrador	Memorial University of Newfoundland
Verna Ryan	Prince Edward Island	Health PEI
Cristina Tassone	Ontario	Cancer Care Ontario
Stacy Taylor	New Brunswick	New Brunswick Department of Health
Lori Wozney	Nova Scotia	Nova Scotia Health Authority

APPENDIX 3: ORGANIZATIONS INCLUDED IN GREY LITERATURE SCAN

Mental health organizations

Canadian Mental Health Association

Canadian Network for Mood and Anxiety Treatments

depressionhurts.ca (Mood Disorders Society of Canada)

ementalhealth.ca (Children's Hospital of Eastern Ontario)

Mental Health Commission of Canada

Mood Disorders Society of Canada

National Survivor User Network (United Kingdom)

Ontario Centre of Excellence for Child and Youth Mental Health

Ontario Shores Centre for Mental Health Sciences

Portico Network

Schizophrenia Society of Canada

Youth Mental Health Canada

Medical organizations or hospitals

Anxiety and Depression Association of America

British Medical Association

Canadian Federation of Mental Health Nurses

Canadian Medical Association

Centre for Effective Practice

College of Physicians and Surgeons of Alberta

Mayo Clinic

Registered Nurses Association of Ontario

SE Health

Client organizations or websites

Arnold P. Gold Foundation

Canadian MPN Group

Canadian Patient Safety Institute

Canadian Public Health Association

Canadian Virtual Hospice

Choosing Wisely Canada

Consumers Health Forum of Australia

familydoctor.org (American Academy of Family Physicians)

Health Consumers Alliance of South Australia

Here to Help BC

Medivizor (United States)

Mental Elf (United Kingdom)

myhealth.alberta.ca (Alberta Health Services)

Ontario Lung Association

Open Arms Advocacy

Patient Commando

Patient Power (United States)

patient.info (United Kingdom)

Patients Advisor Network

Patients Canada

The Change Foundation

Us TOO (United States)

Governmental organizations

Agency for Healthcare Research and Quality (United States)

Canada Health Infoway

Canadian Health Coalition

Centers for Disease Control and Prevention (United States)

Department of Health and Human Services (United States)

Digital Health Canada

Federal Drug Administration (United States)

National Health Service (United Kingdom)

Other

MediaSmarts

PREVNet

camh

Canada Health **Infoway**
